# From access to reserve: antimicrobial resistance among etiological agents of central line-associated bloodstream infections in the view of WHO’s AWaRe antimicrobial spectrum

**DOI:** 10.3205/dgkh000559

**Published:** 2025-06-17

**Authors:** Gargee Anand, Rijhul Lahariya, Ketan Priyadarshi, Asim Sarfraz

**Affiliations:** 1 All India Institute of Medical Sciences, Patna, Bihar, India

**Keywords:** central line-associated bloodstream infections, CLABSI, antimicrobial resistance, WHO AWaRe, intensive care units, antimicrobial stewardship, infection control, pathogen-directed therapy, healthcare associated infection

## Abstract

**Aim:**

Central line-associated bloodstream infections (CLABSI) remain a major contributor to morbidity and mortality in critically ill patients. The rise of antimicrobial resistance (AMR) exacerbates treatment challenges, making it crucial to examine pathogen resistance patterns. This study analyses CLABSI-associated pathogens’ antimicrobial susceptibility using the WHO’s AWaRe antimicrobial framework, providing insights to guide targeted treatment and strengthen infection control strategies.

**Methods:**

This observational study (2021–2024) assessed data from adult and pediatric ICUs to evaluate CLABSI incidence, microbial etiology, and antimicrobial susceptibility trends. We categorized antimicrobials based on the WHO’s AWaRe classification system, analysing their susceptibility to Access, Watch, and Reserve antimicrobials. Statistical analysis was performed using SPSS version 22.

**Results:**

Among 5,398 patient records, 101 cases of CLABSI were confirmed. The predominant pathogens were *Klebsiella (K.) pneumoniae* (27.7%), *Acinetobacter* spp. (19.8%), and *Candida* spp. (17.8%). A worrying decline in susceptibility to Access- and Watch-category antimicrobials was observed in key pathogens. *K. pneumoniae* demonstrated a steep decline in susceptibility to Access-category agents, from 27.8% in 2021 to 16.7% in 2023. Conversely, Reserve-category antimicrobials maintained 100% efficacy across the study period. *Acinetobacter* spp. exhibited resistance to both Access- and Watch-category antimicrobials by 2024. *Pseudomonas aeruginosa* showed a drastic drop in Watch-category susceptibility, from 44.5% in 2021 to 0% in 2023, while Reserve-agents remained effective. These results underline the growing reliance on Reserve antimicrobials and the diminishing effectiveness of first-line agents. Furthermore, a fluctuation in CLABSI rates was also observed, with a significant reduction in infection rates in 2024 after the implementation of enhanced infection control practices.

**Conclusion:**

This study highlights the escalating resistance patterns of CLABSI pathogens, with a consternating decline in Access- and Watch-category antimicrobial efficacy. The AWaRe framework proves invaluable in identifying critical resistance trends, demonstrating the need for targeted antimicrobial stewardship. Prioritizing Access antimicrobials as first-line therapies, guided by local resistance data, can preserve the effectiveness of Reserve agents. A strategic focus on the AWaRe classification, coupled with rigorous infection control and stewardship programs, is essential to combat the rising AMR threat and optimize patient outcomes in critical care settings.

## Zusammenfassung

**Zielsetzung:**

ZVK assoziierte Blutstrominfektionen (CLABSI) sind nach wie vor eine der Hauptursachen für Morbidität und Mortalität bei schwerkranken Patienten. Die zunehmende antimikrobielle Resistenz (AMR) verschärft die Herausforderungen bei der Behandlung, weshalb die Untersuchung von Resistenzmustern der Erreger von entscheidender Bedeutung ist. In der Studie wird die Empfindlichkeit von CLABSI-assoziierten Erregern gegenüber Antibiotika anhand der AWaRe Klassifizierung der WHO analysiert, um Erkenntnisse für eine gezielte Behandlung zu gewinnen und Strategien zur Infektionskontrolle zu stärken.

**Methode:**

In der Beobachtungsstudie (2021–2024) wurden Daten von Intensivstationen für Erwachsene und Kinder ausgewertet, um die Häufigkeit von CLABSI, die mikrobielle Ätiologie und die Entwicklung der Empfindlichkeit gegenüber Antibiotika zu untersuchen. Wir kategorisierten die antimikrobiellen Substanzen auf der Grundlage des AWaRe-Klassifizierungssystems der WHO und analysierten ihre Empfindlichkeit gegenüber Access-, Watch- und Reserve-Antibiotika. Die statistische Analyse wurde mit SPSS Version 22 durchgeführt.

**Ergebnisse:**

Unter 5.398 Patientenakten wurden 101 Fälle von CLABSI bestätigt. Die vorherrschenden Erreger waren *Klebsiella (K.) pneumoniae* (27,7%), *Acinetobacter* spp. (19,8%) und *Candida* spp. (17,8%). Bei den wichtigsten Erregern wurde ein besorgniserregender Rückgang der Empfindlichkeit gegenüber antimikrobiellen Mitteln der Kategorien Access und Watch festgestellt. *K. pneumoniae* wies einen starken Rückgang der Empfindlichkeit gegenüber Mitteln der Kategorie „Access“ auf, von 27,8% im Jahr 2021 auf 16,7% im Jahr 2023. Umgekehrt behielten die antimikrobiellen Mittel der Reservekategorie ihre 100%ige Wirksamkeit über den gesamten Studienzeitraum. *Acinetobacter* spp. wiesen bis 2024 eine Resistenz sowohl gegen antimikrobielle Mittel der Access- als auch der Watch-Kategorie auf. *Pseudomonas aeruginosa* zeigte einen drastischen Rückgang der Empfindlichkeit für die Kategorie Watch von 44,5% im Jahr 2021 auf 0% im Jahr 2023, während die Mittel der Kategorie Reserve wirksam blieben. Die Ergebnisse unterstreichen die zunehmende Abhängigkeit von antimikrobiellen Mitteln der Reserve und die abnehmende Wirksamkeit der First line Mittel. Darüber hinaus beobachteten wir eine Fluktuation der CLABSI-Raten, wobei die Infektionsraten im Jahr 2024 nach der Einführung verbesserter Infektionskontrollverfahren deutlich zurückgingen.

**Schlussfolgerung:**

Die Studie verdeutlicht die eskalierenden Resistenzmuster von CLABSI-Erregern mit einem besorgniserregenden Rückgang der antimikrobiellen Wirksamkeit der Kategorien Access und Watch. Der AWaRe-Rahmen erweist sich als wertvoll für die Identifizierung kritischer Resistenztrends und zeigt die Notwendigkeit eines gezielten antimikrobiellen Stewardships. Die Priorisierung von Access-Antibiotika als Erstlinientherapien, die sich an lokalen Resistenzdaten orientiert, kann die Wirksamkeit von Reserve-Wirkstoffen erhalten. Ein strategischer Fokus auf die AWaRe-Klassifizierung in Verbindung mit rigorosen Infektionskontroll- und Stewardship-Programmen ist unerlässlich, um die steigende AMR-Bedrohung zu bekämpfen und die Therapieergebnisse in der Intensivpflege zu optimieren.

## Introduction

Global prevalence data from the World Health Organization (WHO) indicates that the risk of healthcare-associated infections (HAI) is particularly elevated in intensive care units (ICUs), affecting approximately 30% of ICU patients and resulting in significant morbidity and mortality [1]. The prevalence of HAIs differs markedly between developed and developing nations, with incidence rates of 7% and 10% respectively among hospitalized patients [1]. Among all HAIs, CLABSIs represent a substantial economic burden, with an estimated per-case cost of USD 46,000 [2]. A literature review indicates that CLABSIs significantly extend ICU length of stay, with reported excess hospitalization periods ranging from 2.7 to 48.5 days compared to non-infected patients [3]. CLABSIs not only carry substantial risks of illness and death but also demand more intensive and costly treatments compared to other HAIs, resulting in an exceptionally high burden on both patient care and hospital resources. The collective burden of CLABSIs has been estimated as equivalent to the eigth leading cause of death in the United States [4]. The emergence of antimicrobial resistance (AMR) coupled with biofilm formation on medical devices, particularly vascular catheters, presents significant therapeutic challenges [4]. The WHO developed the AWaRe (Access, Watch, Reserve) classification framework to address escalating AMR concerns while preserving therapeutic efficacy of critical antimicrobials [5]. This classification system strategically categorizes antimicrobial agents into three groups based on their therapeutic importance and resistance potential, and aims to mitigate the global health threat posed by AMR through enhanced surveillance, stewardship, and reduction of inappropriate antimicrobial consumption, strategically categorizing antimicrobials to optimize their use in healthcare settings [5], [6]. The present study presents an in-depth analysis of antimicrobial susceptibility profiles in CLABSI-associated pathogens, leveraging the WHO’s AWaRe classification system to offer novel insights into resistance patterns and inform targeted treatment strategies. Hence, the present study examined three key aspects of CLABSIs: incidence rates, microbial etiology, and antimicrobial susceptibility patterns, analyzed through the WHO’s AWaRe framework, to strengthen the synergy between infection prevention and antimicrobial stewardship programs.

## Methods

### Study design 

This observational cross-sectional study encompassed patients from both adult and pediatric ICUs between 2021 and 2024. Inclusion criteria specified central line placement for more than 2 calendar days. Blood cultures were obtained for microbiological evaluation from patients presenting with clinical signs of bloodstream infection/sepsis. Cases of secondary bloodstream infections were excluded from the analysis. Standardized surveillance definitions of CLABSIs as per Centers for Disease Control and Prevention, National Healthcare Safety Network (CDC, NHSN) were followed [7]. An isolate was classified as multidrug-resistant (MDR) when it was non-susceptible to at least one antimicrobial agent in three or more antimicrobial classes [8]. The CLABSI rate was calculated as: (number of CLABSI/total central line days) ×1,000, expressed as CLABSI per 1,000 central line days [7]. Interpretive breakpoints for antimicrobial susceptibility testing established by CLSI (Clinical and Laboratory Standards Institute), M 100 guidelines for bacterial isolates were used [9]. Relevant data were collected and antimicrobial susceptibility patterns were analyzed using the WHO’s AWaRe classification [5].

### Data collection 

Patient data were retrospectively extracted from two institutional databases: the Hospital Information System (HIS) and HAI surveillance records. The HIS provided microbiological data, including blood culture results and antimicrobial susceptibility profile. HAI surveillance forms were used for gathering demographic information, clinical diagnosis, central line insertion sites, ICU length of stay, mortality/patient outcomes, and daily clinical assessments for catheter-related infection manifestations.

### Patient and public involvement 

In this study, there was no patient or public involvement, as the data were solely collected from the records department.

### Statistical analysis 

All relevant data were entered in a Microsoft Excel 2019 spreadsheet. Normality distribution for all continuous variables was tested using Q-Q plots, histograms, and the Shapiro-wilk test. Continuous variables were expressed using mean (±SD)/ median (IQR) according to their normality, while categorical variables were expressed as percentages/proportions. as appropriate. Bivariate comparison of categorical variables was performed using the Chi-squared test and Fisher’s exact test. Graphs depicting antimicrobial susceptibility trend as per WHO’s AWaRe classification were made using Microsoft Excel 2019. Statistical analysis was conducted using Statistical Package for Social Sciences (SPSS) version 22. A p-value of <0.05 was designated as statistically significant. 

## Results

Over the four-year (2021–2024) study period, records of 5398 patient who met the predefined inclusion criteria were assessed, of whom 101 patients developed CLABSI with Laboratory Confirmed Bloodstream Infection 1 (LCBI 1) criteria as per the CDC, NHSN surveillance criteria. The annual incidence of CLABSI is shown in Table 1 (Tab. 1), and overall incidence of CLABSI is depicted in Table 2 (Tab. 2) by type of ICU.

CLABSI more commonly occurred in patients having femoral access (22 CLABIS/150 femoral line). Analysis revealed a statistically significant predilection for CLABSIs among patients with femoral venous catheterization (p=0.001*). 

Microbiological analysis of CLABSIs revealed a predominance of Gram-negative organisms (76/101; 75.3%), with *Candida* spp. (18/101; 17.8%) and Gram-positive organisms (7/101; 6.9%) comprising the remaining isolates (Table 3 (Tab. 3)).

Analysis of antimicrobial susceptibility patterns across the WHO AWaRe categories revealed distinct temporal trends among isolated pathogens. *K. pneumoniae* exhibited a declining trend in Access-category susceptibility from 27.8% (2021) to 16.7% (2023), with a slight increase to 25.9% in 2024. Watch-category susceptibility showed a marked decrease from 37.5% (2021) to 2.5% (2022), followed by gradual increase to 15.3% (2024). Reserve-category antimicrobials maintained 100% efficacy throughout the study period. *Acinetobacter* spp. demonstrated fluctuating Access-category susceptibility: 22.2% (2022), increasing to 27.8% (2023), before declining to 0% (2024). Watch-category susceptibility showed consistently low rates, peaking at 9.52% (2023). Reserve antimicrobials maintained 100% efficacy from 2022–2024. *E. coli* susceptibility to Access-category antimicrobials decreased from 25% (2023) to 0% (2024), with a parallel decline in Watch-category susceptibility from 8.9% to 0%. However, Reserve-category antimicrobials maintained 100% efficacy. *Burkholderia* spp. maintained consistent Access-category susceptibility (100%) when isolated, while Watch-category susceptibility declined from 83.3% (2021) to 50% (2024). *P. aeruginosa* showed variable Watch-category susceptibility, decreasing from 44.5% (2021) to 0% (2023), with Reserve-category susceptibility declining from 66.7% (2021–2022) to 50% (2023).

**Figure 1:**
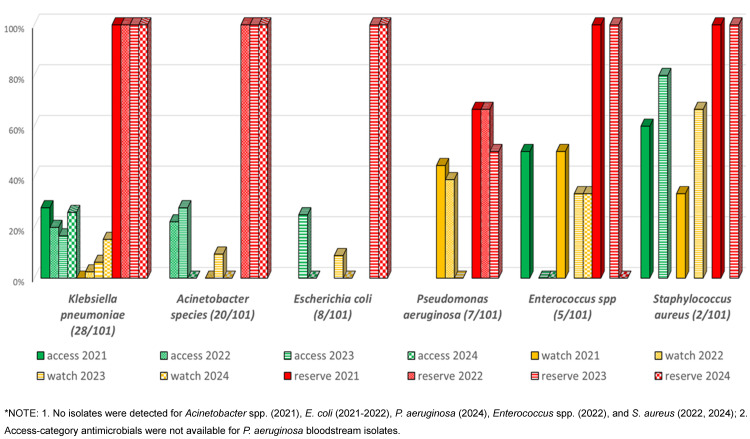
Susceptibility of Access, Watch and Reserve antibiotics among CLABSI isolates by year

Among Gram-positive organisms, *Enterococcus* spp. showed decreasing Access-category susceptibility from 50% (2021) to 0% (2023–2024), with Watch-category susceptibility declining from 50% (2021) to 33.3% (2023–2024). Reserve-category efficacy varied from 100% to 0%. *S. aureus* maintained relatively high Access-category susceptibility (60–80%), with Watch-category susceptibility increasing from 33.3% to 66.7%, and consistent Reserve-category efficacy at 100%. Overall susceptibility of access, Watch- and Reserve-category antimicrobials for various isolated microorganisms among CLABSI patients are shown in Figure 1 (Fig. 1). Annual susceptibility trend (2021–2024) for various antimicrobials among isolated organisms is depicted in Table 4 (Tab. 4).

## Discussion

This groundbreaking study is the first to analyse CLABSI pathogens’ AMR using the WHO’s AWaRe framework, offering critical insights for targeted treatment strategies. Analysis of CLABSI incidence over the four-year surveillance period (2021–2024) revealed notable variations. The baseline CLABSI rate in 2021 was 6.18 per 1,000 central line days, which demonstrated a substantial decline to 1.69 per 1,000 central line days in 2022, representing a 72.7% reduction. However, 2023 witnessed an increase to 3.75 per 1,000 central line days, followed by a subsequent decrease to 2.45 per 1,000 central line days in 2024.

This fluctuation in CLABSI rates warrants careful interpretation. The initial high rate in 2021 can be attributed to the lesser number of ICUs under surveillance and it might reflect the baseline period before implementation of enhanced prevention protocols. Following the elevated CLABSI rates in 2023 (3.75 per 1,000 central line days), implementation of enhanced insertion and maintenance-bundle practices led to a significant reduction in infection rates to 2.45 per 1,000 central line days in 2024, representing a 34.7% decrease. Notably, the central line utilization showed a progressive increase from 2,911 days in 2021 to 10,583 days in 2024, suggesting expanded critical care services or increased patient complexity. This increased device utilization might have contributed to the observed variations in infection rates. 

These findings align with the published literature reporting CLABSI rates of 5 per 1,000 catheter days, while other Indian studies reported CLABSI rates ranging from 0.48 to 27 per 1,000 catheter days in various healthcare settings [10], [11]. The observed temporal variations underscore the dynamic nature of HAIs and emphasize the need for sustained vigilance in prevention strategies. 

Statistical analysis showed a significantly greater predilection for CLABSI occurrence in the presence of femoral catheterization (p-value <0.001*) [12], [13]. The differential risk of CLABSIs across insertion sites can be attributed to anatomical variations, local microbiological colonization patterns, and site-specific mechanical factors. 

The etiological spectrum of CLABSI revealed a predominance of Gram-negative organisms, constituting 75.2% of CLABSIs. Among these, *K. pneumoniae* emerged as the primary pathogen (27.7%), followed by *Acinetobacter* spp. (19.8%). This microbial distribution pattern corresponds with the literature, which documents the predominance of Gram-negative organisms in device-associated bloodstream infections [14].

Antimicrobial susceptibility testing of all CLABSI isolates revealed substantial AMR to first-line agents, a finding that aligns with a previous study [15].

The analysis of antimicrobial susceptibility patterns reveals concerning trends in pathogen resistance profiles across the WHO AWaRe classification framework. Analysis revealed worrying AMR patterns among predominant pathogens, with *K. pneumoniae* showing a progressive decline in Access-category susceptibility (27.8% to 16.7%) and *Acinetobacter* spp. demonstrating complete resistance to both Access- and Watch-categories by 2024. Notably, *P. aeruginosa* exhibited significant resistance development, with Watch-category susceptibility declining from 44.5% to 0% and Reserve-category efficacy decreasing from 66.7% to 50%. Despite these alarming trends, Access-category antimicrobials maintained better susceptibility profiles compared to Watch-category agents for most isolates. The sustained efficacy of Reserve-category antimicrobials (100% susceptibility) among major Gram-negative pathogens, while therapeutically promising, raises concerns about increasing reliance on last-resort antimicrobials. This pattern of escalating resistance to first-line agents, necessitating increased usage of Reserve antimicrobials, underscores the critical need for robust antimicrobial stewardship programs to preserve therapeutic options across all AWaRe categories, hence cascade reporting of antimicrobial susceptibility test (AST) results is of utmost importance.

Among Gram-positive organisms, the decreasing susceptibility of *Enterococcus* spp. to Access- and Watch-category antimicrobials, coupled with variable Reserve-category efficacy, suggests emerging resistance patterns requiring careful monitoring. Conversely, *S. aureus* maintained relatively favourable susceptibility profiles, particularly to Access-category agents, potentially reflecting effective infection control measures.

A paradigm shift is necessary in prescribing practices within ICUs, emphasizing pathogen-directed therapy guided by local susceptibility data rather than defaulting to broad-spectrum Watch- and Reserve-group antimicrobials. Prioritizing Access antimicrobials as first-line therapies, wherever appropriate, will help preserve efficacy of Watch and Reserve agents [16]. The most urgent step needed now is to implement targeted bundle care practices, antimicrobial stewardship strategies aligned with the WHO’s AWaRe classification, even in critical care settings [17], [18].

## Conclusions

The study highlights a precarious situation where the efficacy of Access/Watch antimicrobials is compromised and thus increased reliance is placed on Reserve antimicrobials. This complicates patient management and poses a global health threat by AMR. Thus, prioritizing Access antimicrobials as first-line, where appropriate, will preserve the efficacy of Watch and Reserve agents, mitigating the emergence of extensively drug-resistant strains. This strategy, coupled with

- implementation of pathogen-directed therapy based on local resistance data rather than empiric broad-spectrum antimicrobial use,
- development of targeted antimicrobial stewardship programs aligned with the WHO AWaRe framework, specifically adapted for critical care settingsrigorous infection, and
- control measures and continuous surveillance

offers a promising path to combat AMR in HAIs while adhering to the WHO’s AWaRe even in critical care settings.

## Notes

### Competing interests

The authors declare that they have no competing interests.

### Funding sources

The authors hereby declare that no financial support was received for this study.

### Authors’ ORCIDs

- Anand G: https://orcid.org/0009-0008-0473-389X
- Lahariya R: https://orcid.org/0009-0003-5769-4509
- Priyadarshi K: https://orcid.org/0000-0003-4623-3523
- Sarfraz A: https://orcid.org/0000-0002-6256-7649

## Figures and Tables

**Table 1:**
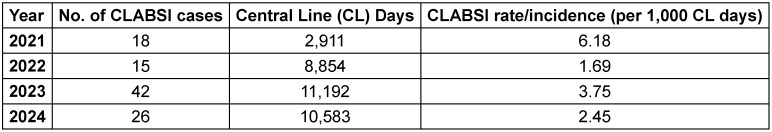
Annual incidence of CLABSI (2021–2024)

**Table 2:**
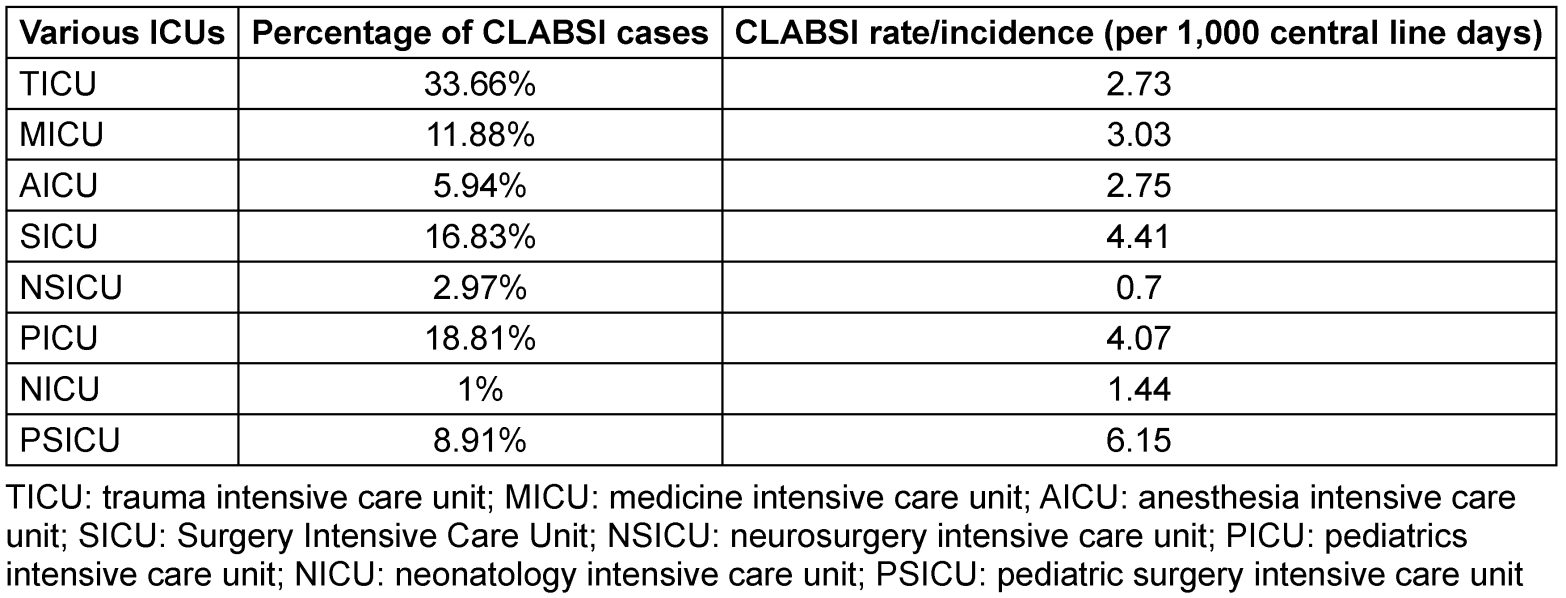
ICU-specific incidence of CLABSI

**Table 3:**
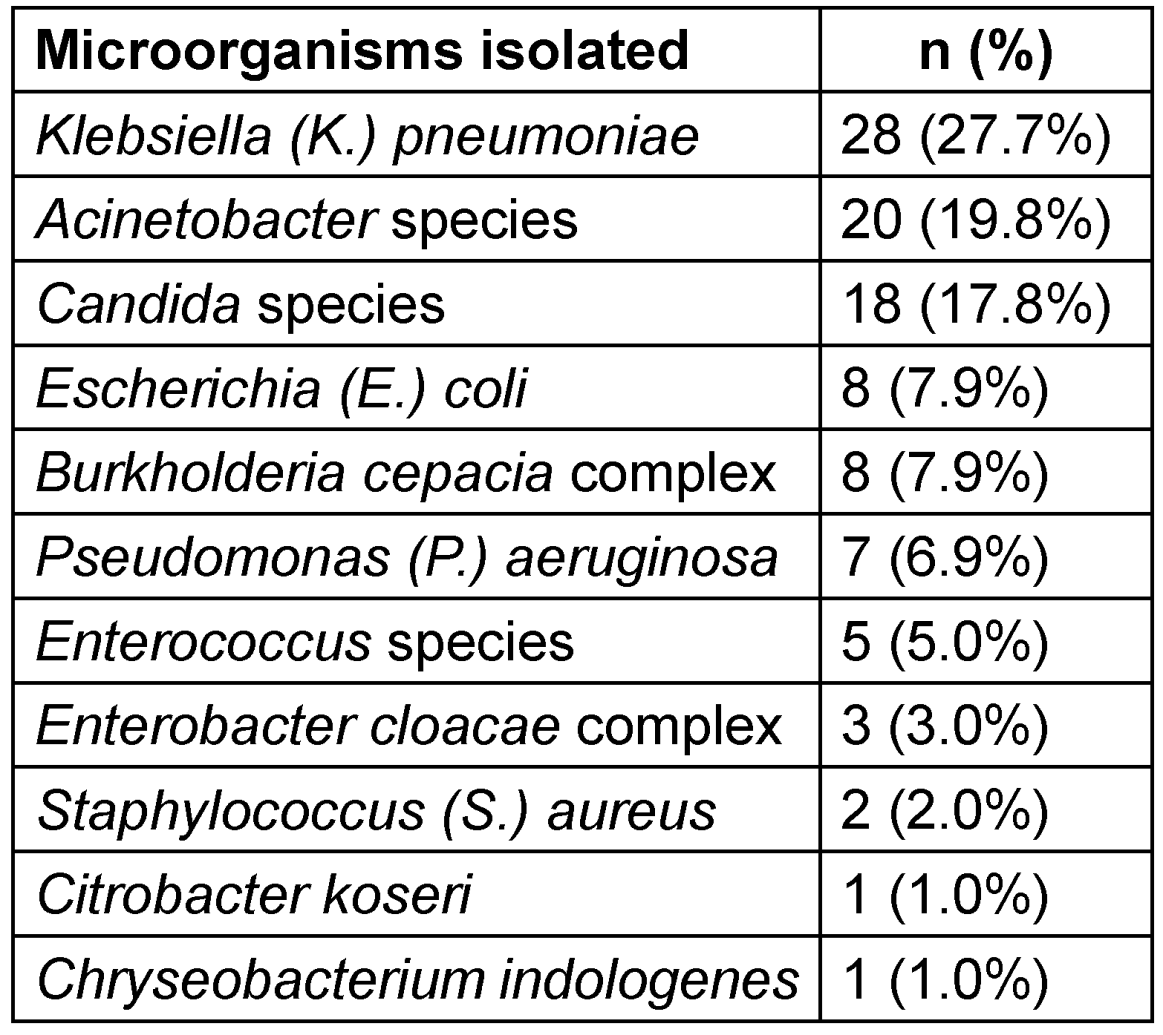
Microorganisms isolated from CLABSI cases (n=101)

**Table 4:**
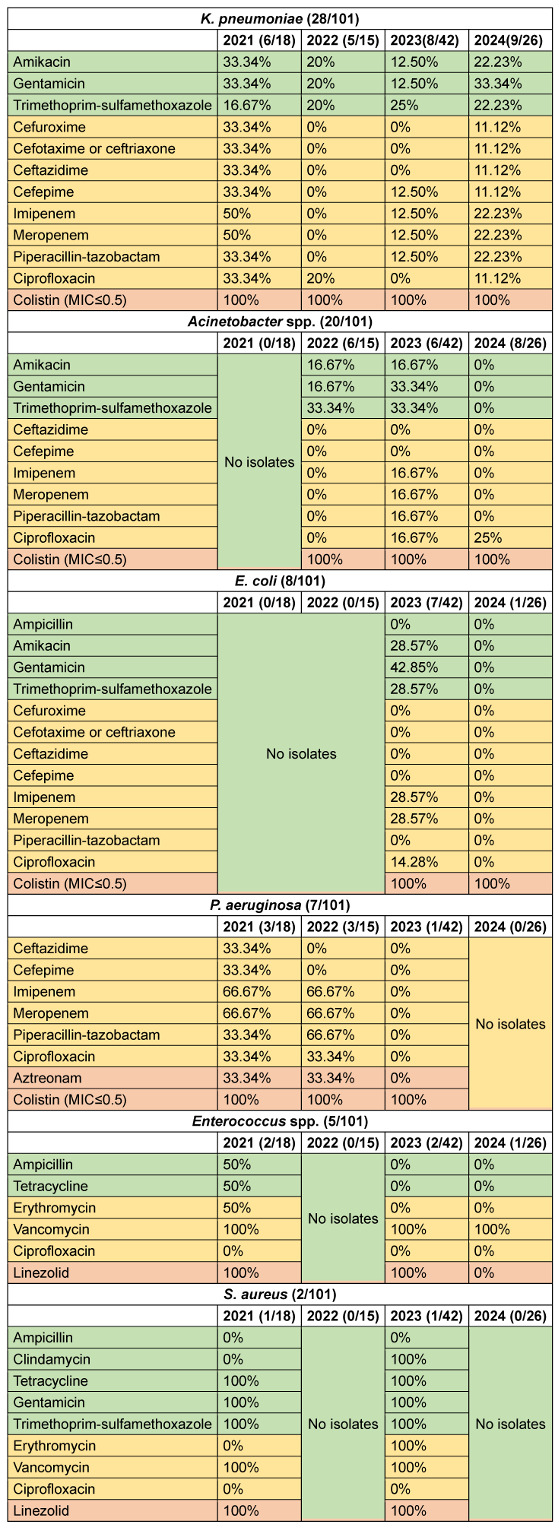
Annual susceptibility trend (2021–2024) of various antibiotics against various CLABSI isolates (CLABSI cases 101)
